# Efficient Europium Sensitization via Low-Level Doping in a 2-D Bismuth-Organic Coordination Polymer

**DOI:** 10.1021/acs.cgd.2c01475

**Published:** 2023-04-05

**Authors:** Alexander C. Marwitz, Anuj K. Dutta, Morgan A. McDonald, Karah E. Knope

**Affiliations:** Department of Chemistry, Georgetown University, Washington, District of Columbia 20057, United States

## Abstract

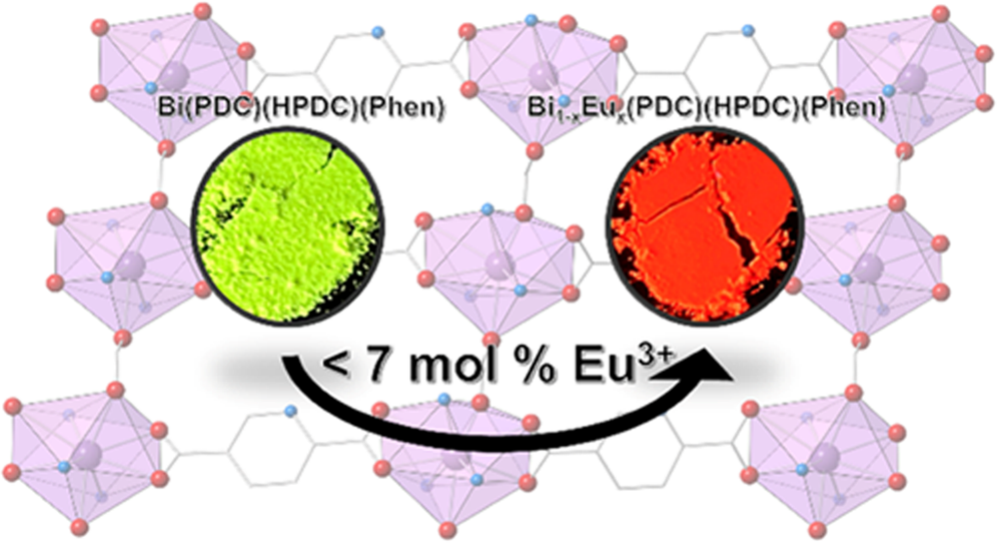

A new bismuth-organic compound containing 1,10-phenanthroline (phen) and 2,5-pyridinedicarboxylic acid (PDC) was synthesized and structurally characterized by single-crystal X-ray diffraction. The structure consists of 2-D {Bi(phen)(HPDC)(PDC)}*_n_* sheets wherein the PDC ligands bridge metal centers via three unique bonding modes. The 2-D sheets are further connected through strong hydrogen-bonding interactions to form a 3-D supramolecular network. The parent compound displayed yellow photoluminescence in the solid state at room temperature. Doping studies were undertaken to incorporate Eu^3+^ into the structure, statistically replacing Bi^3+^ in small quantities (1, 5, and 10 mol % Eu^3+^ relative to Bi^3+^). All three compounds displayed characteristic Eu^3+^ emission, with total quantum yields as high as 16.0% and sensitization efficiencies between 0.21 and 0.37 depending on the Eu^3+^ doping percentage.

## Abstract 2

A bismuth-organic coordination polymer, Bi(PDC)(HPDC)(Phen), that is built from pyridinedicarboxylate and phenanthroline ligand sets was synthesized and characterized. The homometallic compound exhibits yellow luminescence. Low-level doping of Eu^3+^ into the metal-organic phase via site substitution results in red emission, with quantum yields approaching 16%.

## Introduction

Bismuth-based materials have exhibited promising applications in medicine,^1−5^ catalysis,^6−9^ and luminescence.^10−15^ Bismuth is well known for its nontoxicity, in part due to its poor solubility, which lends itself to biological applications. Furthermore, it is relatively globally abundant and cheap, particularly when compared to other metals commonly used in luminescent materials design such as iridium, platinum, or ruthenium.^16^ Apart from these considerations, bismuth affords unique electronic and structural handles that have been harnessed toward various properties.^17^ With respect to photoluminescent materials, early work by Vogler and colleagues proposed that Bi^3+^ and the other main group metals with *n*s^2^ electron configurations (e.g., Sb^3+^, Pb^2+^, Sn^2+^) could undergo similar photochemistry and metal-to-ligand charge transfer (MLCT) transitions in the visible region as those displayed by d^10^ metal ions.^18,19^ Consistent with this notion, Bi^3+^ has recently proven to be a promising candidate for luminescent materials. For example, in 2010 and 2011, zur Loye and colleagues published a series of solid-state coordination polymers containing Bi^3+^ and 2,5-pyridinedicarboxylic acid (2,5-PDC) that displayed visible photoluminescence, with examples of blue, green, and even white light emission.^20−22^ More recent examples of bismuth-organic coordination polymers have similarly exhibited blue, green, white, and yellow emission attributed to charge transfers, intraligand transitions, and s → p transitions of bismuth.^23−27^ Bismuth-organic compounds have also displayed exciting properties including mechanochromism,^28,29^ solvochromism,^30^ and polymorphism-dependent emission.^31,32^ Furthermore, due to the similar oxidation states, ionic radii, and coordination geometries of Bi^3+^ and Ln^3+^ metal ions, bismuth-organic materials have been utilized as hosts for the trivalent lanthanides. Doping of Ln^3+^ ions into bismuth-organic materials has yielded the highly desirable emissive properties of Ln^3+^ ions while using just a fraction of the expensive rare earth starting materials relative to a homometallic Ln^3+^ compound.^33−43^

Still the development of structure–property relationships in photoluminescent bismuth-organic compounds remains relatively understudied. Yet the attractive properties of those compounds reported motivates further development of structure–property relationships in this class of materials. Previously, we reported on a series of bismuth halide organic compounds that displayed metal-halide to ligand charge transfer (XMLCT) transitions.^42^ In this work, a correlation was found between the extent of stereochemical activity of the 6s^2^ lone pair (lp), the Bi–X bond length, and the energy of the highest occupied molecular orbital (HOMO), giving new insight into the effect of 6s^2^ lone pair stereochemical activity on optical properties. More recently, we reported on the first example of 1,10-phenanthrolinium (Hphen) phosphorescence in the solid state at room temperature from a 2,6-pyridinedicarboxylic acid-bridged (2,6-PDC) bismuth dimer with Hphen in the outer coordination sphere.^43^ It was found that the strong supramolecular interactions (hydrogen bonding, π–π interactions, and lp−π interactions) provided by the bismuth dimeric unit effectively stabilized the triplet state of the outer coordination sphere fluorophore leading to long-lived phosphorescence.

Inspired by this work, we set out to design a multidimensional framework utilizing a dual-ligand system of 2,5-PDC and 1,10-phenanthroline (phen) to probe the effect of a second coordinating, and π-stacking, fluorophore on the structure and photoluminescence of Bi-PDC materials. A 2D-coordination polymer, Bi(HPDC)(PDC)(Phen), was synthesized and displayed yellow photoluminescence upon UV irradiation. This material was found to be amenable to Eu^3+^ incorporation; Eu^3+^ doping studies were undertaken, and the resulting materials displayed intense Eu^3+^ emission with no evidence of the original yellow luminescence attributed to the bismuth-organic host. Quantum yields and sensitization efficiencies of the Eu^3+^ emission are reported.

## Results and Discussion

### Structure Descriptions

**Figure 1:**
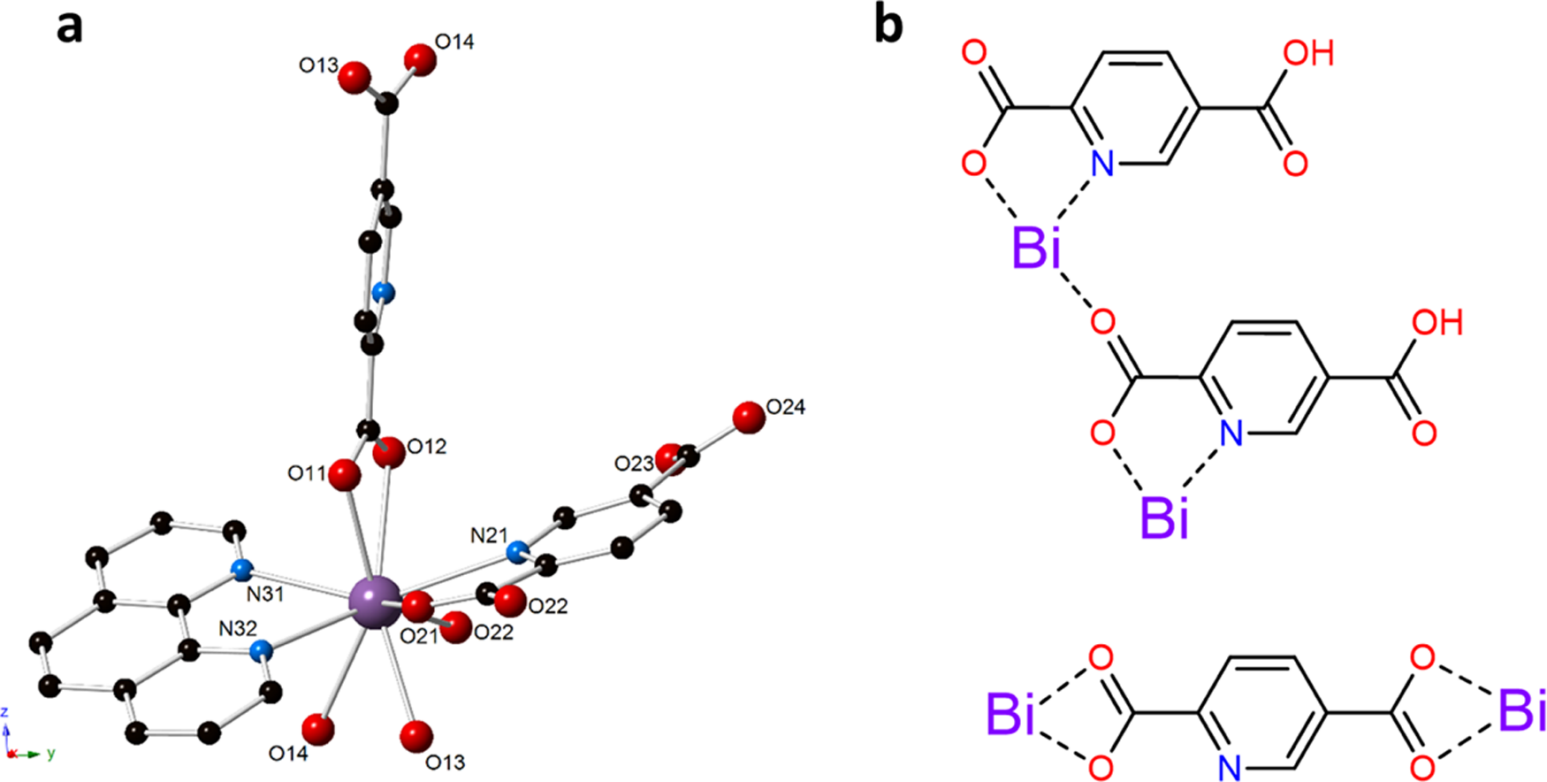
(a) Ball and stick representation of the local coordination environment of Bi in **Bi-1**. Purple = bismuth, blue = nitrogen, red = oxygen, and black = carbon atoms. Hydrogen atoms have been omitted for clarity. (b) Illustration of the three unique coordination modes of Bi-2,5-PDC in **Bi-1**.

The local structure of **Bi-1** consists of a nine-coordinate bismuth metal center. Bismuth is coordinated to a bidentate phenanthroline, two symmetry-equivalent bidentate PDCs that chelate through the carboxylate oxygen atoms (O11, O12, O13, O14), and two symmetry-equivalent HPDCs. While one of the HPDCs binds through a single carboxylate oxygen (O22) (Figure 1), the other exhibits bidentate coordination through a nitrogen (N21) and a carbonyl oxygen (O21). The Bi–O distances range from 2.357(2) to 2.812(2) Å, and the Bi–N distances range from 2.491(2) to 2.614(2) Å. The high coordination number of bismuth coupled with the lack of significant asymmetry in the Bi–O and Bi–N bond lengths suggests the 6s^2^ lone pair is stereochemically inactive and the metal center is holodirected.

**Figure 2:**
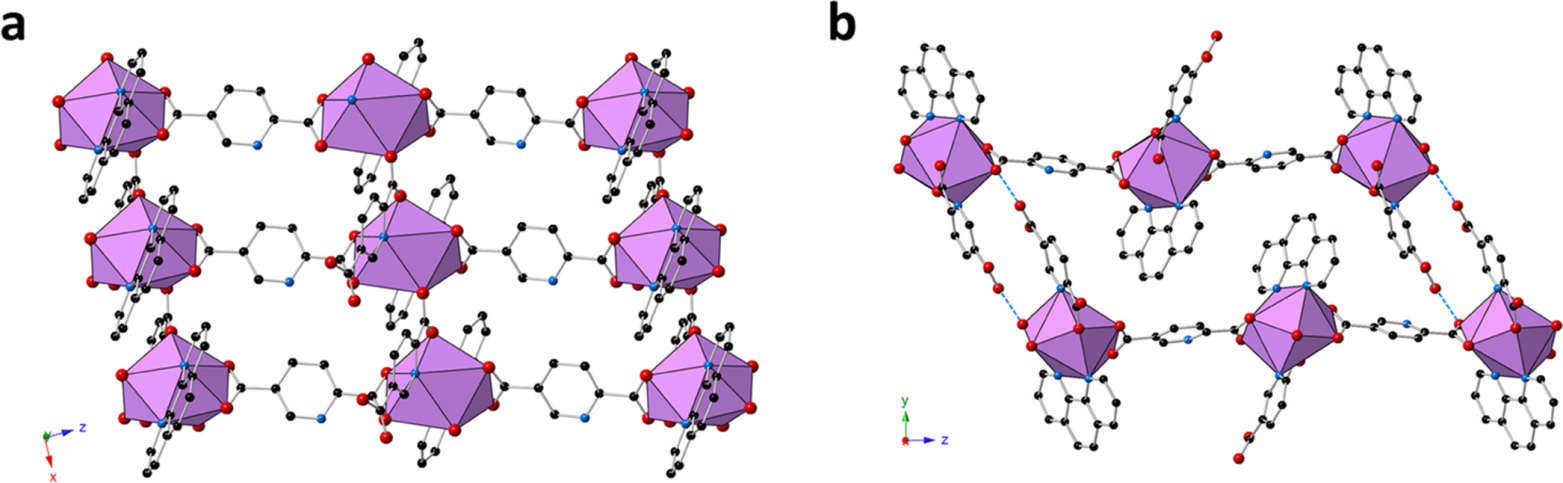
Polyhedral representation of **Bi-1** viewed down: (a) [010] showing the extended 2D network that consists of BiO_6_N_3_ polyhedra bridged through PDC units, (b) [100] highlighting the 3D supramolecular network that results from hydrogen-bonding interactions. Hydrogen-bonding interactions between neighboring sheets in (b) are shown with blue dashed lines. Purple = bismuth, blue = nitrogen, red = oxygen, and black = carbon atoms. Hydrogen atoms have been omitted for clarity.

As shown in Figure 2a, the bismuth metal centers are connected through two crystallographically distinct PDC units, one bound through two carboxylate O atoms and the other bound through one carboxylate O and the N from the pyridine ring, to form {Bi(phen)(HPDC)(PDC)}*_n_* 2-D sheets that extend in the {010} plane. Additionally, π–π interactions exist between phens bound to neighboring (bridged) metal centers, down the [100], with centroid···centroid distances (C_Phen_···C_Phen_) of 3.643(1) Å and slip angles of 22.9° (Figure S2). The Bi---Bi distances are 6.1068(3) Å. The sheets are further connected to one another through hydrogen-bonding interactions between the singly protonated HPDC on one sheet and the doubly deprotonated PDC on another sheet, resulting in a 3-D supramolecular structure, with an O–H···O distance of 2.650(2) Å and O–H···O angle of 168(3)° (Figure 2b).

### Europium Doping

The limited stereochemical activity of the 6s^2^ lone pair of the Bi together with the bound phen in **Bi-1** suggested that Eu doping may be promising both from synthetic and materials properties perspectives. As such, three Eu-doped compounds, **Bi**_**0.99**_**Eu**_**0.01**_**-1**, **Bi**_**0.95**_**Eu**_**0.05**_**-1**, and **Bi**_**0.90**_**Eu**_**0.10**_**-1**, were synthesized using 1, 5, and 10 mol % Eu relative to Bi in the synthesis, respectively. Phase purity was confirmed by PXRD. Attempts to synthesize phases with higher levels of Eu incorporation, including a 50 mol % Eu compound, resulted in significant phase separation with impurities visible in the reaction vessel. Structural analysis of the impurity revealed a previously reported homometallic Eu(PDC)(HPDC) coordination polymer.^44,45^ Notably, efforts to prepare an Eu-only analogue similarly resulted in the formation of the homometallic Eu(PDC)(HPDC) phase. These results are consistent with previous work from our group that has shown that despite similarities in Bi and Eu charge and ionic radii, solubility and reaction kinetics often limit Eu doping.^33^

To confirm the incorporation of Eu^3+^ in **Bi**_**0.99**_**Eu**_**0.01**_**-1**, **Bi**_**0.95**_**Eu**_**0.05**_**-1**, and **Bi**_**0.90**_**Eu**_**0.10**_**-1** via site substitution of Bi^3+^, emission spectra were collected on single crystals using a Raman microscope with an excitation source of 532 nm (Figures S9–S15). Harmonic peaks of the ^5^D_0_ → ^7^F_1_ transition of Eu^3+^ dominated the spectra between 1750 and 3000 cm^–1^ for the Eu-doped materials. Moreover, spectra were collected for **Bi**_**0.90**_**Eu**_**0.10**_**-1** at various focal depths. The ratios of the Eu^3+^ harmonic peak at 1860 cm^–1^ and the Bi-organic vibrational peak at 1593 cm^–1^ (Table S1) were relatively constant, consistent with the homogeneous incorporation of Eu^3+^ within the crystal. Additional support for Eu^3+^ doping in the crystal structure rather than at the crystal surface was provided by leaching experiments. Crystals of the doped compound, **Bi**_**0.90**_**Eu**_**0.10**_**-1**, were soaked in water, the reaction solvent. After 24 h, the mother liquor was filtered, diluted with 3% HNO_3(aq)_, and checked for Eu and Bi isotopes via ICP-MS. No signals for either Eu or Bi were detected, suggesting Eu^3+^ does not simply reside at the crystal surface.

ICP-MS was performed on the Eu-doped samples to quantify the amount of Eu that was incorporated into the structures (Table 1). The amount of Eu^3+^ in the samples is significantly less than the relative percentage added to the reaction during synthesis. Such variability in the doping percent has been observed for other bismuth-organic compounds,^43^ and suggests the coordination environment around the metal center is one of several factors that dictates the level of Ln^3+^ incorporation. Bi^3+^ has a [Xe]4f^14^5d^10^6s^2^ electron configuration and the 6s^2^ lone pair can be stereochemically active or inactive. This can lead to coordination environments ranging from higher-coordinate, holodirected spherical geometries, to lower-coordinate, hemidirected geometries with an open coordination site trans to the shortest bond to the bismuth. The trivalent lanthanides, however, more commonly display isotropic coordination spheres. Thus, it is reasonable to assume that a holodirected bismuth center, such as that in **Bi-1**, may improve the level of lanthanide incorporation into a bismuth host by promoting site substitution. However, despite the relatively similar coordination environments, it is also important to note that Eu^3+^ and Bi^3+^ vary in solubility and this may be the origin of the disparity between the synthetic and experimental Eu^3+^ mol %.

**Table 1:** Percent Eu Doping for **Bi**_**1–*x***_**Eu***_**x**_***-1** by ICP-MS

compound	mol % Eu^3+^ added	mol % Eu^3+^ (ICP-MS)
**Bi**_**0.99**_**Eu**_**0.01**_**-1**	1	0.280 ± 0.002
**Bi**_**0.95**_**Eu**_**0.05**_**-1**	5	3.053 ± 0.017
**Bi**_**0.90**_**Eu**_**0.10**_**-1**	10	6.166 ± 0.039

### Photoluminescence

**Figure 3:**
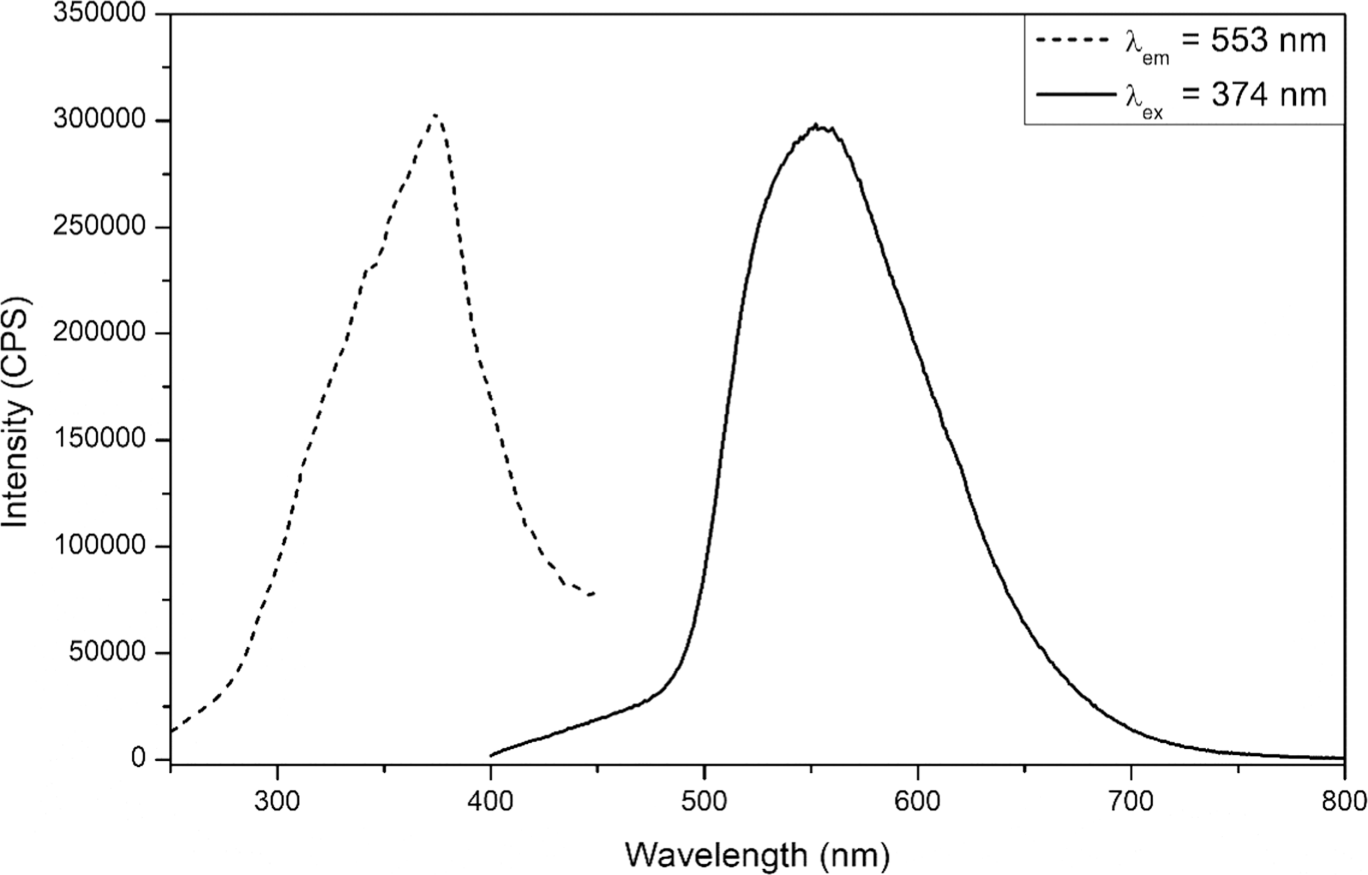
Excitation (dashed line; λ_em_ = 553 nm) and emission (solid line; λ_ex_ = 374 nm) spectra for **Bi-1**.

The parent compound, **Bi-1**, displayed yellow luminescence upon UV irradiation. As shown in Figure 3, upon excitation at 374 nm, the compound exhibits a broad emission with the maximum intensity centered at 553 nm. The emissive lifetime was determined from a phosphorescence decay spectrum that was fit with a triple-exponential decay function (Figure S17). The lifetimes were 676.9, 78.7, and 7.1 μs. The longer lifetime is likely attributed to emission from a triplet charge transfer state, ^3^MLCT. Yellow and orange emissions have been previously observed for bismuth-organic compounds with the emission attributed to an MLCT or XMLCT.^19,29,46^ The 78.7 μs lifetime may be attributed to a triplet intraligand transition, and the 7.1 μs lifetime most likely results from residual 2,5-PDC fluorescence or scattering from the xenon lamp used for the lifetime measurement.

Incorporation of even small amounts of Eu^3+^ into **Bi-1** results in characteristic Eu^3+^ transitions with emission from **Bi-1** no longer discernable. The Eu^3+^-doped phases have an excitation maximum of 350 nm, with a small shoulder in the excitation spectra at 374 nm, the λ_max_ of excitation for the undoped sample (Figure S16). Lanthanide 4f–4f transitions are Laporte forbidden, leading to a low molar absorptivity. To get around this, researchers often exploit the antenna effect, wherein an organic fluorophore is used to sensitize the excited state of the Ln^3+^ from the ligand T_1_ state, resulting in Ln^3+^ emission. In the case of **Bi**_**0.99**_**Eu**_**0.01**_**-1**, **Bi**_**0.95**_**Eu**_**0.05**_**-1**, and **Bi**_**0.90**_**Eu**_**0.10**_**-1**, phen is likely acting as the sensitizer for Eu^3+^ emission. Previous studies on Eu^3+^ compounds containing phen have attributed excitation wavelengths around 350 nm to absorption and sensitization by phenanthroline, consistent with this work.^47−49^ The small shoulder in the excitation spectra at 374 nm for the Eu-doped compounds is attributed to the ^3^MLCT excited state from the undoped compound, which likely sensitizes Eu^3+^ emission to a small degree. The low intensity of Eu^3+^ emission upon excitation at 374 nm may be due to poor energy matching of the ^3^MLCT excited state and the Eu^3+^ emitting level.

**Figure 4:**
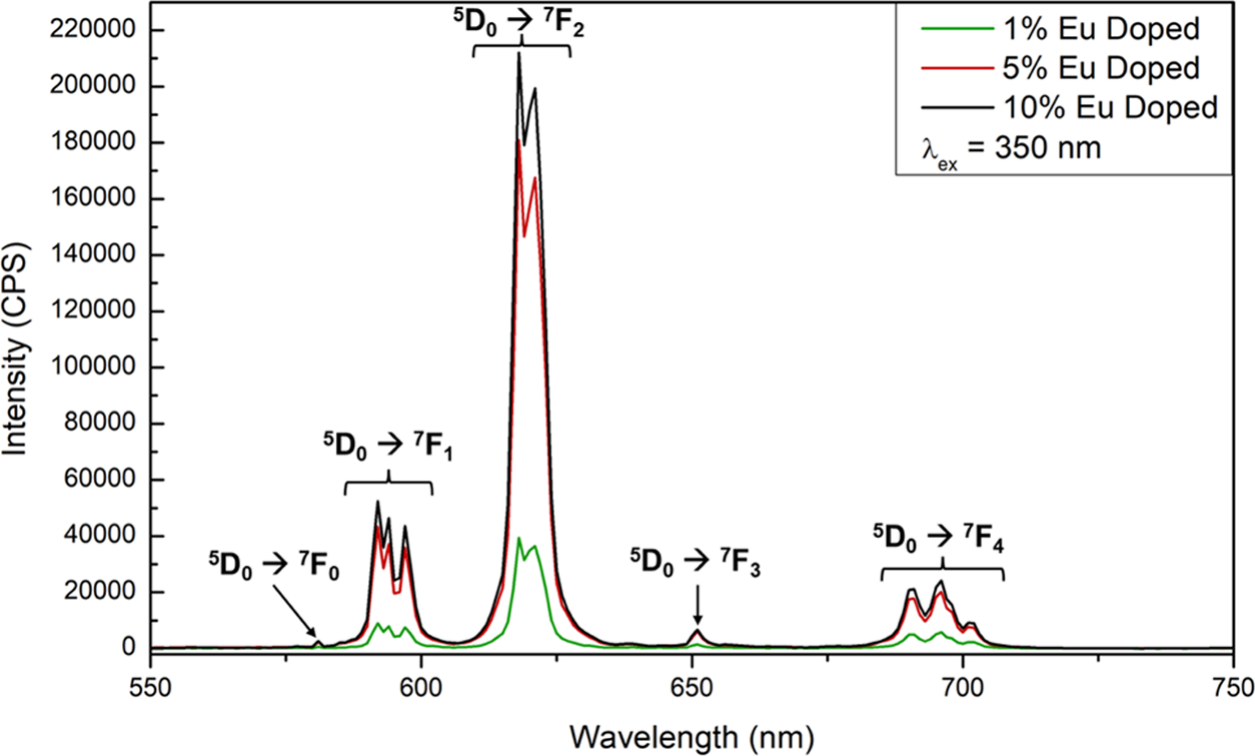
Emission spectra for **Bi**_**0.99**_**Eu**_**0.01**_**-1** (green), **Bi**_**0.95**_**Eu**_**0.05**_**-1** (red), and **Bi**_**0.90**_**Eu**_**0.10**_**-1** (black) upon excitation at 350 nm.

The emission spectra for **Bi**_**0.99**_**Eu**_**0.01**_**-1**, **Bi**_**0.95**_**Eu**_**0.05**_**-1**, and **Bi**_**0.90**_**Eu**_**0.10**_**-1** are shown in Figure 4. Eu^3+^ emission results from the ^5^D_0_ → ^7^F_J_ transitions of Eu^3+^, where J = 0–6 (although the ^5^D_0_ → ^7^F_5_ and ^7^F_6_ transitions are often not visible). For the compounds reported herein, the ^5^D_0_ → ^7^F_0_ transition is evidenced as a very small peak at 580 nm. The magnetic dipole transition, ^5^D_0_ → ^7^F_1_, is observed starting at 590 nm, with significant splitting resulting in three resolved peaks. The hypersensitive ^5^D_0_ → ^7^F_2_ transition exhibits two resolved peaks of nearly equal intensity at 618 and 621 nm, with much greater intensity than the other transitions. The emissive lifetime was measured for the ^5^D_0_ → ^7^F_2_ transition for all three compounds and yielded lifetimes of 1200, 1198, and 1201 μs in order of increasing Eu^3+^ concentration (Figures S18–S20). Long lifetimes on the order of 10^–3^ s, such as those exhibited by the doped compounds, are consistent with the incorporation of lanthanide into the structure as opposed to at the crystal surface where quenching effects would be expected to yield shorter lifetimes.^50,51^ The ^5^D_0_ → ^7^F_3_ transition is located at 651 nm with too low intensity to reliably determine splitting, and the ^5^D_0_ → ^7^F_4_ transition is observed at 688 nm and shows significant splitting. No evidence of the ^5^D_0_ → ^7^F_5_ and ^7^F_6_ transitions is observed.

The splitting of peaks for the trivalent lanthanides, and specifically Eu^3+^, has been well studied and the extent of splitting is attributed to the site symmetry of the metal center.^52^ In low-symmetry environments (i.e., C_1_, C_i_, etc.), emission from ^2S+1^L_J_ transitions shows significant splitting, to the effect of 2J+1, due to reduced or removed degeneracy between crystal-field levels. The significant splitting observed in the ^5^D_0_ → ^7^F_1_ and ^5^D_0_ → ^7^F_4_ transitions suggests that the symmetry around Eu^3+^ in these compounds is low. This is consistent with the crystal structure: if the Eu^3+^ is site-substituting at the Bi^3+^ site, which has C_1_ site symmetry, the ^5^D_0_ → ^7^F_1_ transition should show three nondegenerate states. This is observed experimentally; however, the ^5^D_0_ → ^7^F_2_ transition should show five nondegenerate states, but only two peaks are seen experimentally. Additional peaks are likely poorly resolved among the significant peak intensity. In fact, deconvolution of the ^5^D_0_ → ^7^F_2_ transition for **Bi**_**0.90**_**Eu**_**0.10**_**-1** is consistent with the presence of five peaks. Therefore, the splitting of the Eu^3+^ emission peaks is consistent with site substitution at Bi^3+^ sites.

### Quantum Yields and Sensitization Efficiencies

Quantum yield measurements were collected for the four compounds to better understand the efficiency of sensitization. **Bi-1** displayed a quantum yield less than 1% and is therefore excluded. As shown in Table 2, the total quantum yield (ϕ_Total_) increases as the concentration of Eu^3+^ increases, although not linearly. This increase in ϕ_Total_ is expected as the excited states that are responsible for sensitizing the Eu^3+^ emission should still be populating in the absence of Eu^3+^. Thus the photons absorbed by the compound should remain relatively constant; however, the number of photons emitted by Eu^3+^ should increase relative to Eu^3+^ concentration. The intrinsic metal-centered quantum yield, ϕ_Eu_, however is a function of the measured lifetime and the radiative lifetime of Eu^3+^. The latter is dependent upon the spontaneous emission probability of the ^5^D_0_ → ^7^F_1_ transition, the refractive index of the medium, and the relative area of emission from the magnetic dipole transition, ^5^D_0_ → ^7^F_1_, to the total area of emission, all of which are effectively constant in these compounds. Thus, ϕ_Eu_ remains constant between the samples. The sensitization efficiency, η_sens_, increases linearly with ϕ_Total_ for the same reason—the donor excited states are able to more efficiently transfer energy to the Eu^3+^ emissive states when saturated with Eu^3+^ ions.

**Table 2:** Photophysical Measurements for Eu-Doped Compounds, **Bi**_**0.99**_**Eu**_**0.01**_**-1**, **Bi**_**0.95**_**Eu**_**0.05**_**-1**, and **Bi**_**0.90**_**Eu**_**0.10**_**-1**, as well as Other Reported Eu-Doped Bismuth-Organic Compounds^33−35^

compound	ϕ_total_	ϕ_Eu_	τ (μs)	η_sens_	reference
**Bi**_**0.99**_**Eu**_**0.01**_**-1**	0.093(10)	0.44	1200	0.21	this work
**Bi**_**0.95**_**Eu**_**0.05**_**-1**	0.113(16)	0.44	1198	0.26	this work
**Bi**_**0.90**_**Eu**_**0.10**_**-1**	0.160(6)	0.43	1201	0.37	this work
**Hpy[Bi**_**0.99**_**Eu**_**0.01**_**(TDC)**_**2**_**(H**_**2**_**O)]·1.5H**_**2**_**O**	0.016(2)	0.172	354(4)	0.087	(33)
**(Hpy)**_**2**_**[Bi**_**0.99**_**Eu**_**0.01**_**(TDC)**_**2**_**(HTDC)]·0.36H**_**2**_**O**	0.033(2)	0.297	640(2)	0.116	(33)
**Bi**_**0.97**_**Eu**_**0.03**_**NO**_**3**_**(TTA)**_**2**_**(terpy)**	0.052(9)	0.44	664(44)	0.12	(34)
**(Terpy)Bi**_**0.84**_**Eu**_**0.16**_**(κ**^**2**^**-TC)**_**3**_**·0.47H**_**2**_**O**	0.255	0.509	1216	0.501	(35)

Quantum yield measurements for the parent compound, **Bi-1**, were less than 1% and thus excluded.

To assess the quantum yields and sensitization efficiencies of **Bi**_**0.99**_**Eu**_**0.01**_**-1**, **Bi**_**0.95**_**Eu**_**0.05**_**-1**, and **Bi**_**0.90**_**Eu**_**0.10**_**-1**, they were compared to three classes of relevant materials: other heterometallic Eu-doped bismuth-organic host materials, homometallic Eu-phen compounds, and homometallic Eu-2,5-PDC compounds. While there are numerous examples of lanthanide doping into bismuth-organic host materials, only three publications reported values for Eu^3+^ quantum yields and η_sens_ (included in Table 2).^33−35^ In 2021, we reported a series of Eu-doped heteroleptic bismuth-organic compounds with thenoyltrifluoroacetone (TTA) and 2,2′;6′,2″-terpyridine (terpy) that showed relatively inefficient Eu^3+^ emission with only one compound displaying a ϕ_Total_ > 1%.^34^ The most efficient compound contained 2.9 mol % Eu^3+^ in the structure, comparable to **Bi**_**0.95**_**Eu**_**0.05**_**-1** reported herein, yet only displayed a ϕ_Total_ of 0.052(9) and an η_sens_ of 0.12. Here, even the compound with the lowest Eu incorporation, **Bi**_**0.99**_**Eu**_**0.01**_**-1**, eclipses that with a ϕ_Total_ of 0.093(10) and an η_sens_ of 0.21. Two other reports from our group in 2018 showed similar results.^33,35^ One sample, Bi(2-thiophenecarboxylate)_3_(terpy), which contained 16 mol % Eu^3+^, displayed greater ϕ_Total_ and η_sens_ than **Bi**_**0.90**_**Eu**_**0.10**_**-1**.

Structures consisting of Eu^3+^ and phen are relatively common; 367 crystal structures containing Eu and phen are reported in the Cambridge Structural Database (CSD) Version 5.43 as of June 2022.^53^ These structures show a variety of reported efficiencies; in one example containing phen and 3-phenyl-4-benzoyl-5-isoxazolone (HPBI), the authors report a solid-state ϕ_Total_ of 0.11 and an η_sens_ of 0.20.^54^ Another heteroleptic phase with phen and 1,3-bis(4,4,4-trifluoro-1,3-dioxobutyl)phenyl (BTP) exhibited significantly higher efficiencies with a ϕ_Total_ of 0.65 and an η_sens_ of 0.83.^55^ Eu-2,5-PDC compounds are far less numerous than Eu-phen. The solid-state quantum yield of only one structure is reported, with a ϕ_Total_ value of 0.21.^56^ By comparison, the three Eu-doped compounds, **Bi**_**0.99**_**Eu**_**0.01**_**-1**, **Bi**_**0.95**_**Eu**_**0.05**_**-1**, and **Bi**_**0.90**_**Eu**_**0.10**_**-1**, exhibit moderate efficiencies that lie between those previously reported for phen- or 2,5-PDC-containing Eu^3+^ compounds; this is significant given the low concentrations of Eu^3+^ in these materials.

Efficiency in Eu^3+^ materials is in large part dictated by the T_1_ state energy of the sensitizing ligand. An ideal sensitizer should have a T_1_ energy approximately 2000–5000 cm^–1^ above the emitting level of the lanthanide.^49,52^ As mentioned previously, the excitation maximum of 350 nm in the Eu-containing compounds is consistent with previous Eu-phen structures, suggesting phen is acting as the sensitizer for **Bi**_**0.99**_**Eu**_**0.01**_**-1**, **Bi**_**0.95**_**Eu**_**0.05**_**-1**, and **Bi**_**0.90**_**Eu**_**0.10**_**-1**. Furthermore, phen is reported to have a T_1_ energy of 21,480 cm^–1^, approximately 4000 cm^–1^ above the ^5^D_0_ emitting level of Eu^3+^ (17,500 cm^–1^).^49^ That, coupled with the rigidity induced by the bismuth-organic coordination polymer, is likely the source of the relatively high efficiency in the reported materials.

## Conclusions

A novel heteroleptic bismuth-organic coordination polymer, Bi(HPDC)(PDC)(Phen), was synthesized hydrothermally using 1,10-phenanthroline and 2,5-pyridinedicarboxylic acid. The parent compound, **Bi-1**, displayed yellow emission at room temperature in the solid state. Three europium-doped analogues were synthesized with varying concentrations of Eu^3+^. These doped compounds displayed solely Eu^3+^ emission with the least efficient of the three showing a total quantum yield and sensitization efficiency of 0.093(10) and 0.21, respectively, while only containing 0.280 mol % Eu^3+^. The efficiencies of these materials were found to be greater than reported values for all but one Eu-doped bismuth-organic compound, and greater than other Eu(2,5-PDC) compounds. Overall, this work shows that efficient Ln^3+^ emission can be achieved in certain bismuth-organic hosts, using low concentrations (as low as 0.280 mol %) of Ln^3+^ ions.

## Experimental Section

### Materials

Bi(NO_3_)_3_·5H_2_O (Fisher, 99.2%), 2,5-pyridinedicarboxylic acid (Tokyo Chemical 98%), 1,10-phenanthroline (Alfa Aesar 99%), and Eu(NO_3_)_3_·6H_2_O (Beantown Chemical 99.9%) were used as received. Nanopore water (≤0.05 μS; Millipore) was used in all experiments.

### Synthesis

#### Bi(2,5-HPDC)(2,5-PDC)(Phen) (**Bi-1**)

2,5-Pyridinedicarboxylic acid (0.250 g, 1.500 mmol), Bi(NO_3_)_3_·5H_2_O (0.0395g, 0.100 mmol), and 1,10-phenanthroline (0.0360 g, 0.20 mmol) were added to a 23 mL Telfon-lined Parr autoclave and diluted with 5 mL of nanopore water. The Parr autoclave was placed in an oven at 140 °C for 72 h, then allowed to slowly cool over 6 h. Clear, yellow rods that emitted yellow upon UV exposure (λ_Ex_ = 365 nm) were isolated as a phase-pure product after rinsing with water and ethanol. Product yield: 46.7% based on bismuth. Elemental analysis for C_26_H_15_BiN_4_O_8_: Calcd (Obs.): C, 43.35% (43.19%); H, 2.10% (2.09%); N, 7.78% (7.78%).

#### Bi_0.99_Eu_0.01_(2,5-HPDC)(2,5-PDC)(Phen) (**Bi**_**0.99**_**Eu**_**0.01**_**-1**)

**Bi**_**0.99**_**Eu**_**0.01**_**-1** was synthesized using the same procedure as **Bi-1**, adding a 0.1 mL aliquot of a 0.01 M Eu(NO_3_)_3_ aqueous solution to the reaction mixture. Clear, colorless rods that emitted red under a UV lamp were obtained. Product yield = 82.3% based on bismuth. Elemental analysis for C_26_H_15_Bi_0.997_Eu_0.003_N_4_O_8_ (Bi:Eu obtained from ICP-MS): Calcd (Obs.): C, 43.36% (42.99%); H, 2.10% (1.97%); N, 7.78% (7.73%).

#### Bi_0.95_Eu_0.05_(2,5-HPDC)(2,5-PDC)(Phen) (**Bi**_**0.95**_**Eu**_**0.05**_**-1**)

**Bi**_**0.95**_**Eu**_**0.05**_**-1** was synthesized using the same procedure as **Bi**_**0.99**_**Eu**_**0.01**_**-1**, but instead adding a 0.5 mL aliquot of a 0.01 M Eu(NO_3_)_3_ aqueous solution. Clear, colorless rods that emitted red under a UV hand lamp were isolated. Product yield = 65.1% based on bismuth. Elemental analysis for C_26_H_15_Bi_0.97_Eu_0.03_N_4_O_8_ (Bi:Eu obtained from ICP-MS): Calcd (Obs.): C, 43.45% (43.11%); H, 2.10% (1.94%); N, 7.78% (7.80%).

#### Bi_0.90_Eu_0.10_(2,5-HPDC)(2,5-PDC)(Phen) (**Bi**_**0.90**_**Eu**_**0.10**_**-1**)

**Bi**_**0.90**_**Eu**_**0.10**_**-1** was synthesized using the same method as **Bi**_**0.99**_**Eu**_**0.01**_**-1**, but instead adding a 1.0 mL aliquot of a 0.01 M Eu(NO_3_)_3_ aqueous solution. Clear, colorless rods that exhibited red luminescence were obtained as a pure phase. Product yield = 64.5% based on bismuth. Elem anal. Elemental analysis for C_26_H_15_Bi_0.94_Eu_0.06_N_4_O_8_ (Bi:Eu obtained from ICP-MS): Calcd (Obs.): C, 43.56% (43.42%); H, 2.11% (2.01%); N, 7.81% (7.87%).

### Structure Determination

A single crystal of **Bi-1** was isolated from the bulk, placed in N-paratone, and mounted on a MiTeGen micro mount. Single-crystal X-ray diffraction data were collected at 100(2) K. Details of the data collection and processing, as well as refinement details are provided in the Supporting Information. Crystallographic structure refinement details are reported in Table 3.

**Table 3:** Structure Refinement Details for **Bi-1**

	(Bi-1)
MW (g/mol)	2881.60
*T* (K)	100
λ (K α)	0.71073
μ (mm^–1^)	7.512
crystal system	monoclinic
space group	**P**21/**n**
*a* (Å)	6.1068(3)
*b* (Å)	17.3299(8)
*c* (Å)	22.3844(10)
α (°)	90
β (°)	92.5680(10)
γ (°)	90
volume (Å^3^)	2366.57(19)
*Z*	4
*R*_int_	0.0387
*R* (*I* > 2σ)	0.0190
*w*R**_2_	0.0401
GooF	1.091
residual density max and min (e/Å^3^)	0.749 and −0.880
CCDC No.	2217273

### Characterization Methods

Powder X-ray diffraction data (Figures S3 and S4) were collected on the reaction products that yielded single crystals of **Bi-1** and the Eu-doped analogues using a Rigaku Ultima IV X-ray diffractometer. Patterns were collected from 3 to 40° in 2θ with a step speed of 1°/min using Cu Kα radiation (λ = 1.542 Å). Combustion elemental analysis data were collected on a PerkinElmer model 2400 elemental analyzer. The thermal behavior of the compounds was assessed under N_2_ using a TA Instruments Q50 thermogravimetric analyzer with a 5 °C/min temperature ramp rate (Figures S5–8).

### Inductively Coupled Plasma-Mass Spectrometry

ICP-MS data were collected using an Agilent 7800 ICP-MS spectrometer in order to quantify the Bi/Eu ratio of the Eu-doped samples (**Bi**_**0.99**_**Eu**_**0.01**_**-1**, **Bi**_**0.95**_**Eu**_**0.05**_**-1**, and **Bi**_**0.90**_**Eu**_**0.10**_**-1**). Approximately 10 mg of each sample was dissolved in 5% HNO_3_ and diluted to the ppb range. A calibration curve was made for Eu^3+^ using seven standard concentrations (0, 25, 50, 75, 100, 125, and 150 ppb). All ICP-MS solutions were prepared with 5% HNO_3_. The Eu^3+^ concentration was calculated from the resulting calibration curve.

### Photoluminescence

Excitation and emission spectra, lifetimes, and quantum yields for bulk samples of **Bi-1** and the Eu-doped compounds were collected on a Horiba PTI QM-400 fluorometer. Details of the sample preparation and data collection are provided in the Supporting Information. Emission spectra were also collected on single crystals of **Bi-1**, **Bi**_**0.99**_**Eu**_**0.01**_**-1**, **Bi**_**0.95**_**Eu**_**0.05**_**-1**, and **Bi**_**0.90**_**Eu**_**0.10**_**-1** using a Horiba LabRAM HR Evolution Raman spectrometer equipped with a 532 nm excitation source. Spectra were collected using 5% laser power between 300 and 3000 cm^–1^ with 20 accumulations.

## Supporting Information Available

Additional crystallographic details, thermal ellipsoid plots, additional supramolecular packing diagrams, powder X-ray diffraction patterns, thermogravimetric analysis data, Raman spectra, photoluminescence settings and quantum yield sample preparation, photoexcitation spectra, phosphorescence decay plots, and a table of surpramolecular interactions (PDF)
