# Supplementary material for: Efficient Europium Sensitization via Low-Level Doping in a 2-D Bismuth-Organic Coordination Polymer
Source: Cryst Growth Des. 2023 Apr 5;23(5):3330–7. doi: 10.1021/acs.cgd.2c01475 (PMC10950293; doi:10.1021/acs.cgd.2c01475)
Supplement: Supplementary file 1 — cg2c01475_si_001.pdf [file cg2c01475_si_001.pdf]

Efficient Europium Sensitization via Low-Level Doping in a 2-D Bismuth-Organic Coordination Polymer

*Alexander C. Marwitz, Anuj K. Dutta, Morgan A. McDonald, Karah E. Knope**

Department of Chemistry, Georgetown University, Washington, D.C. 20057, United States of America

Supporting Information

I. Additional Crystallographic Details..………………………………………………………….2
II. Packing Diagram Highlighting Supramolecular Structure…….……………………………..4
III. Powder X-Ray Diffraction Patterns..…………………………………………………..…...5
IV. Thermogravimetric Analysis.…………………………………………………………….....6
V. Emission Spectra for Single crystals of Bi-1 and Eu-Doped Compounds collected using a Raman spectrometer ……………………………….………………………………..……….10
VI. Photoluminescence Lifetime and Quantum Yield Data Collection……………..................16
VII. Excitation Spectra for Eu Doped Samples..…………………………………………….....17
VIII. Lifetime Measurements…………………………………………………………..……..18
IX. Supramolecular Interactions……………………………………………………….……22
X. References………………………………………………………………………………23

I. Additional Crystallographic Details

Single crystal X-ray diffraction data was collected on a Bruker D8 Quest Diffractometer equipped with a Photon 100 detector and an IuS X-ray source (Mo Kα radiation; λ = 0.71073 Å). The data were integrated using the SAINT software package included with APEX3.[1-2] An absorption correction was applied using the multi-scan technique in SADABS.[3] The structure was solved using intrinsic methods via SHELXT and refined through full-matrix least squares on F2 using the SHELXL software in shelXle64.[4-5] The resulting CIF can be found as additional supplemental information. Crystallographic data was deposited in the Cambridge Crystallographic Data Centre (CCDC) and may be found at http://www.ccdc.cam.ac.uk/ by referencing number 2217273.

All crystallographic details can be found in the refinement section of the crystallographic information file (CIF). Single crystal X-ray diffraction was not employed to characterize the Eu-doped compounds. For **Bi-1**, all non-hydrogen atoms were refined anisotropically after locating in the Fourier difference map. The single hydrogen involved with hydrogen bonding (on O24) was located in the difference map. The O-H distance in the carboxylic acid was allowed to freely refine and the distance was not fixed. U_{eq} values for the H atom on the carboxylic acid was assigned as 1.5 times its carrier atom; remaining H atom U's were assigned as 1.2 times carrier U_{eq}.

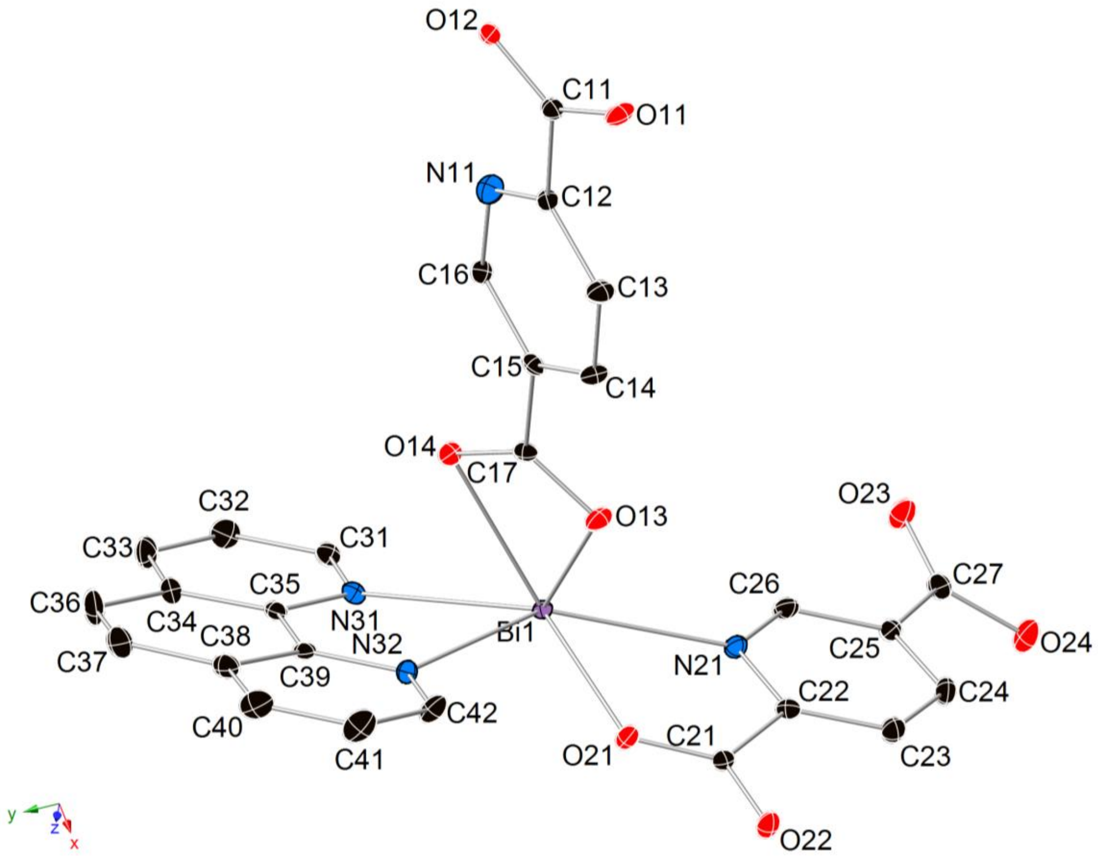

Figure S1. Thermal ellipsoid plot for the asymmetric unit of **Bi-1** at 100 K. Ellipsoids are shown at 50% probability. Hydrogen atoms are not shown for clarity. Purple = bismuth; red = oxygen; blue = nitrogen; black = carbon atoms.

II. Packing Diagram Highlighting Supramolecular Structure

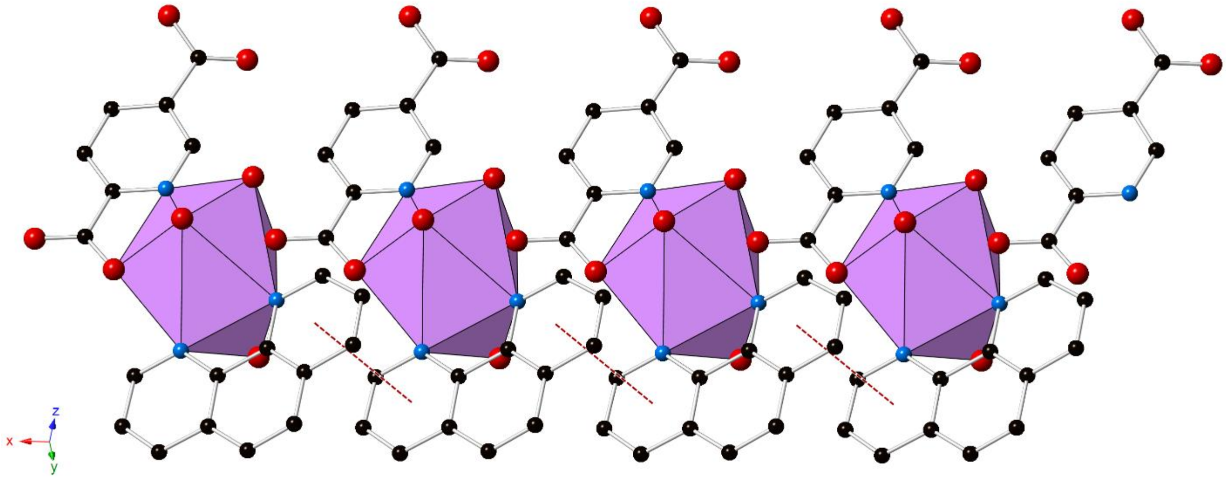

Figure S2. Polyhedral representation of **Bi-1** highlighting the π-π stacking interactions (red dashed lines) between neighboring phens along [100].

III. Powder X-ray Diffraction Patterns

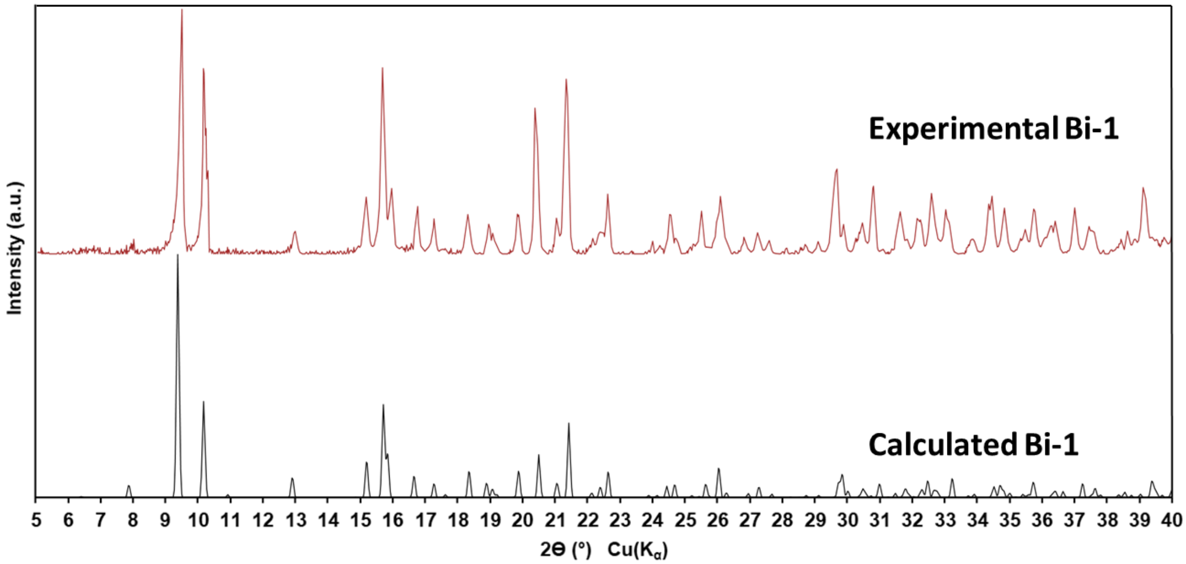

Figure S3. Experimental Powder X-ray Diffraction pattern for **Bi-1** (red) overlaid with the calculated pattern from the single crystal data collected at 100 K (black).

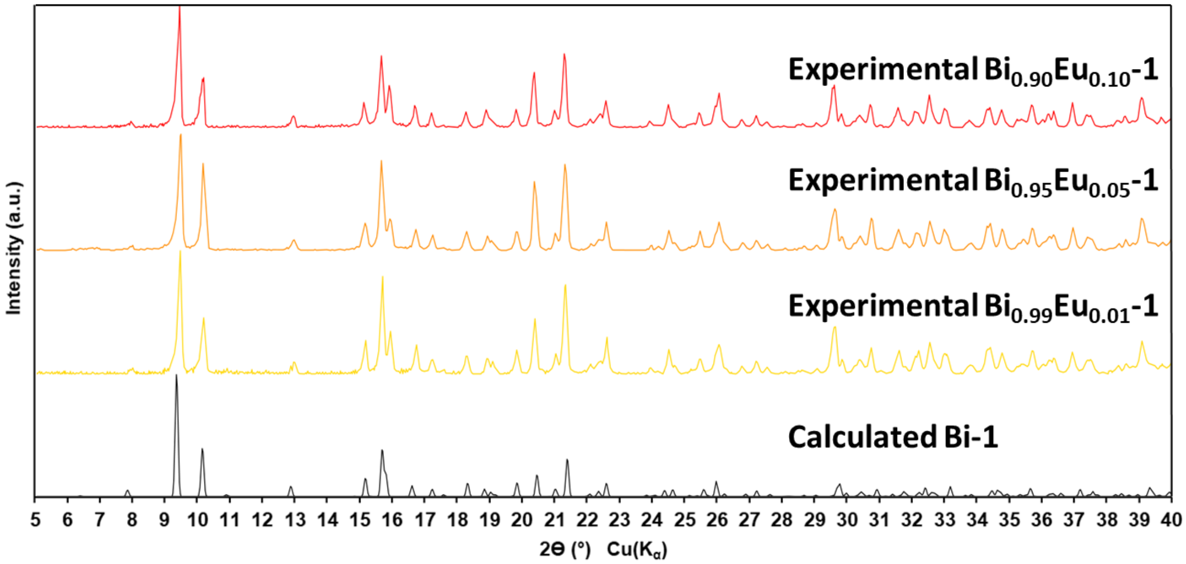

Figure S4. Experimental Powder X-ray Diffraction pattern for **$Bi_{0.99}Eu_{0.01}$-1** (yellow), **$Bi_{0.95}Eu_{0.05}$-1** (orange), and **$Bi_{0.90}Eu_{0.10}$-1** (red) overlaid with the calculated pattern for **Bi-1** from the single crystal data collected at 100 K (black).

IV. Thermogravimetric Analysis

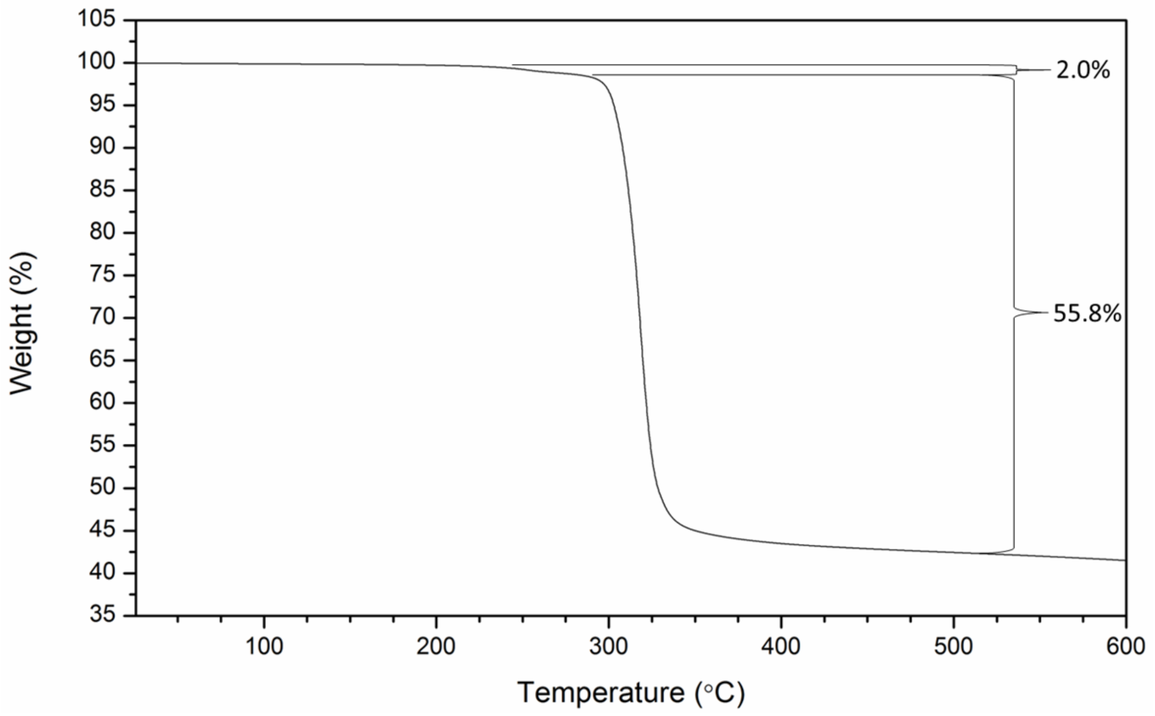

Figure S5. Annotated thermogravimetric analysis of **Bi-1** collected from 30-600 °C at a rate of 5 °C per minute. The compound shows good thermal stability up until ~300 °C.

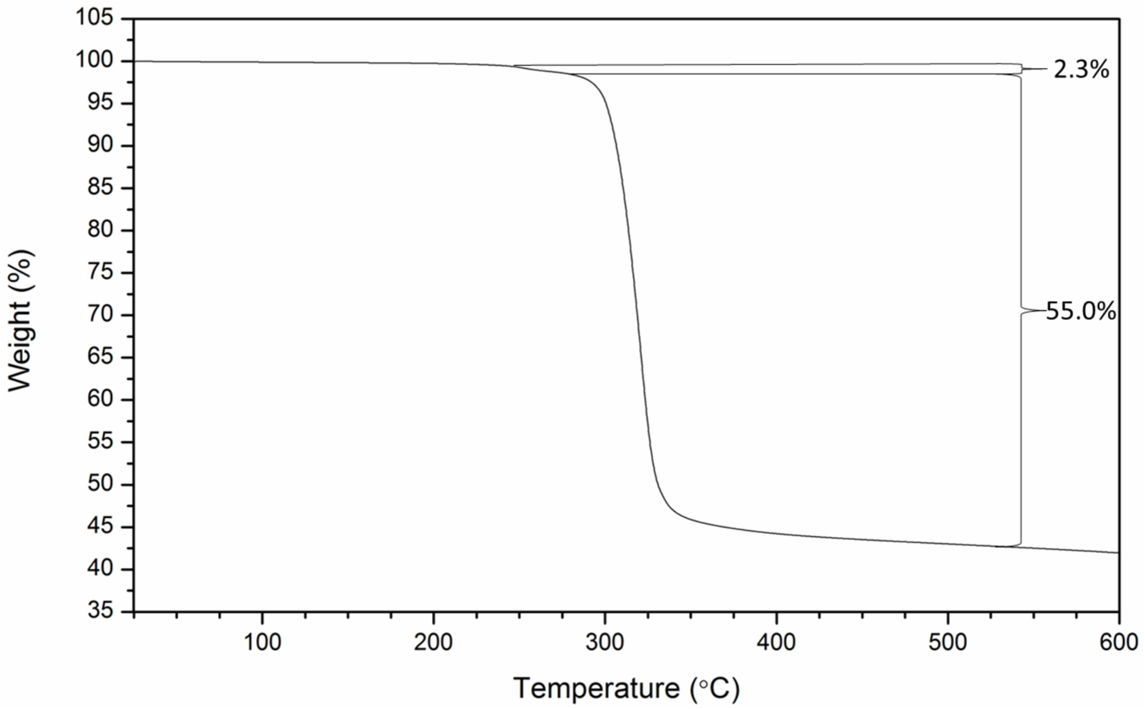

Figure S6. Annotated thermogravimetric analysis of **$Bi_{0.99}Eu_{0.01}$-1** collected from 30-600 °C at a rate of 5 °C per minute. The compound shows good thermal stability up until ~300 °C.

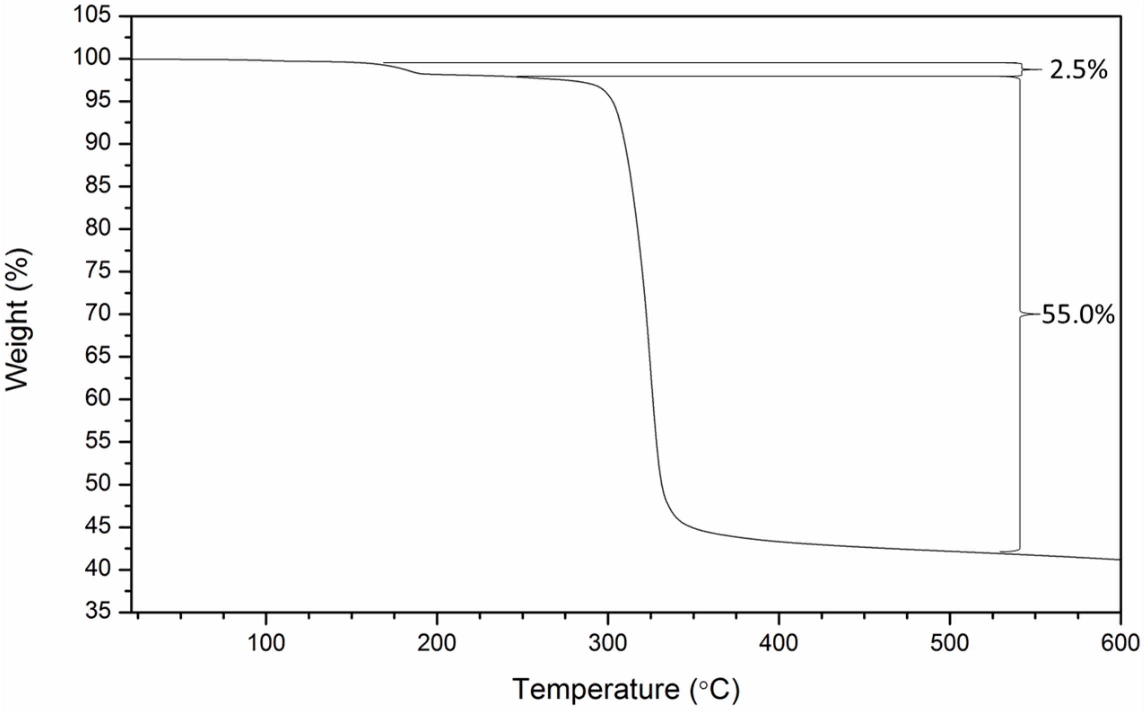

Figure S7. Annotated thermogravimetric analysis of **$Bi_{0.95}Eu_{0.05}$-1** collected from 30-600 °C at a rate of 5 °C per minute. The compound shows good thermal stability up until ~300 °C.

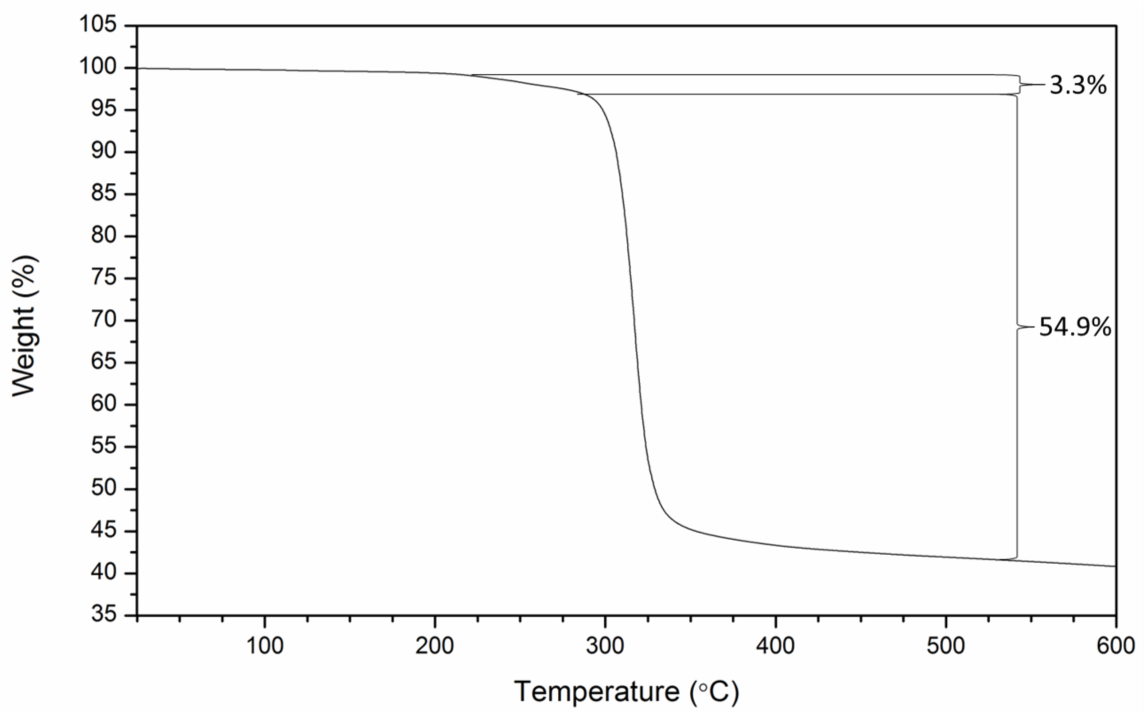

Figure S8. Annotated thermogravimetric analysis of **$Bi_{0.90}Eu_{0.10}$-1** collected from 30-600 °C at a rate of 5 °C per minute. The compound shows good thermal stability up until ~300 °C.

V. Emission Spectra for Single crystals of Bi-1 and Eu-Doped Compounds collected using a Raman spectrometer

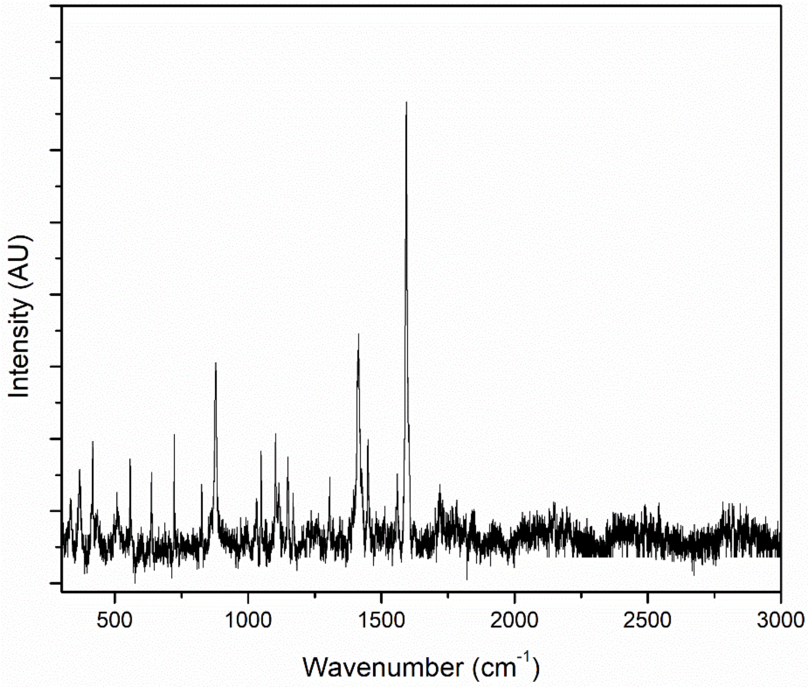

Figure S9. Raman spectra for the undoped sample, **Bi-1**, collected on a single crystal picked from the bulk material.

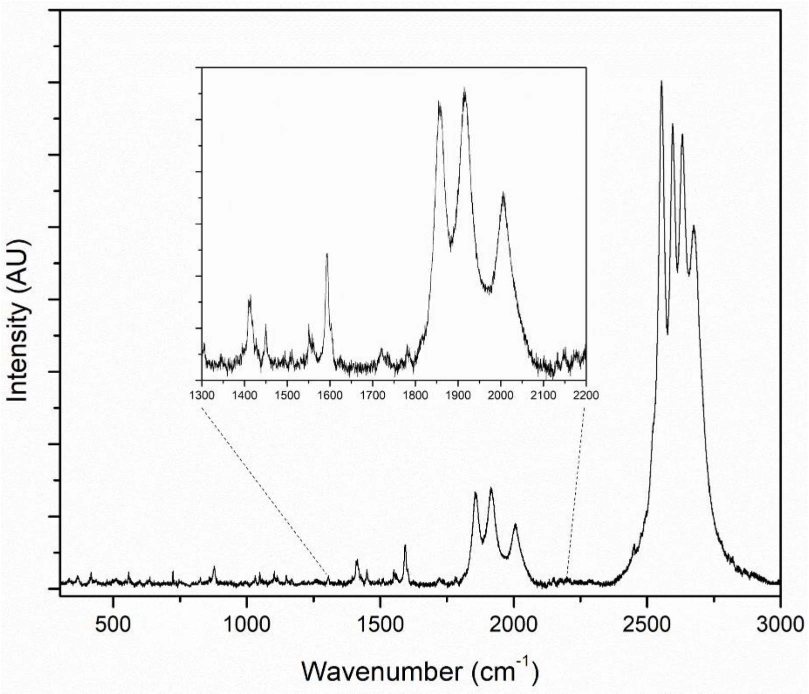

Figure S10. Raman spectra for **$Bi_{0.99}Eu_{0.01}$-1** collected on a single crystal picked from the bulk material. The peaks between 1750 and 3000 cm-1 show various harmonics of the $^5D_0 \rightarrow {}^7F_1$ transition for Eu^{3+}. The inset highlights the region between 1300 and 2200 cm^{-1} for clarity.

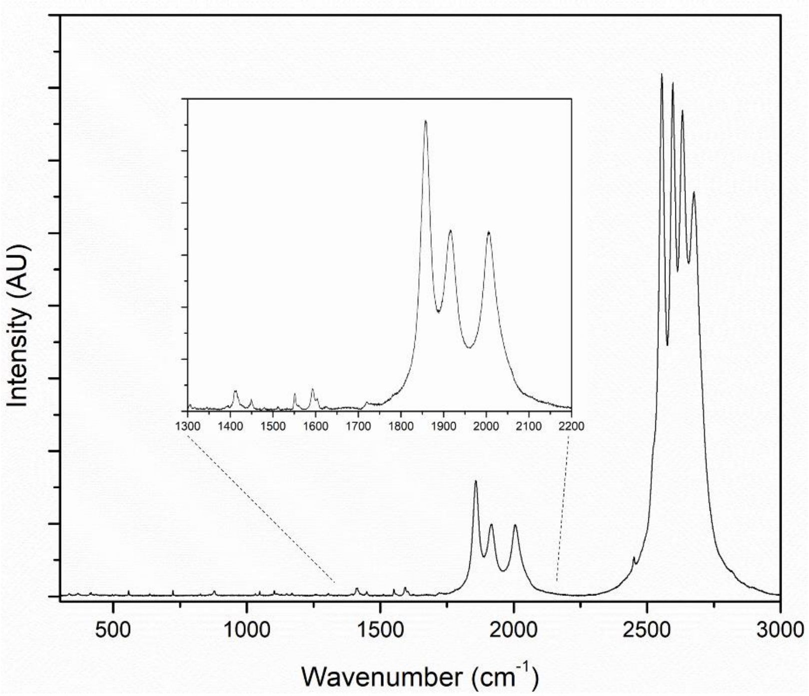

Figure S11. Raman spectra for **$Bi_{0.95}Eu_{0.05}$-1** collected on a single crystal picked from the bulk material. The peaks between 1750 and 3000 cm-1 show various harmonics of the $^5D_0 \rightarrow {}^7F_1$ transition for Eu^{3+}. The inset highlights the region between 1300 and 2200 cm^{-1} for clarity.

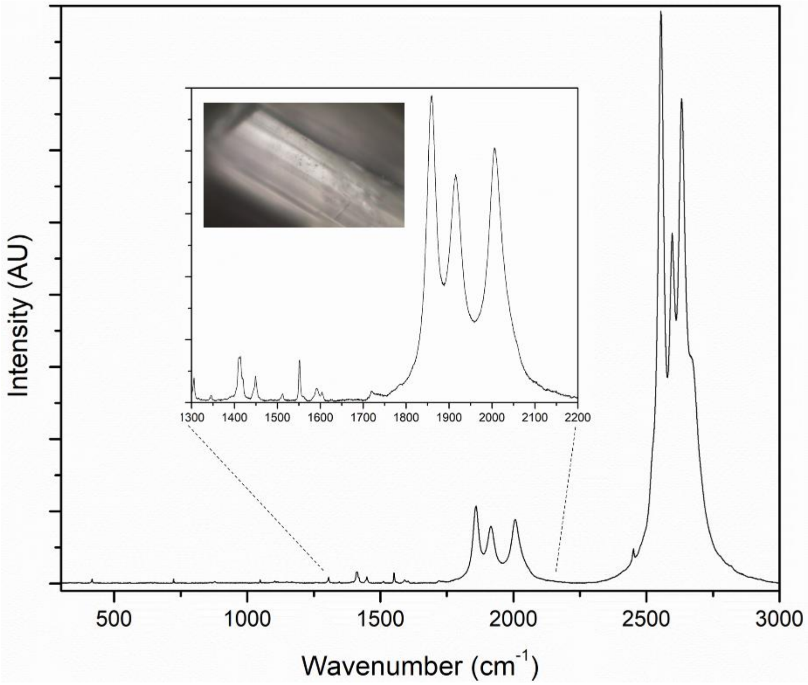

Figure S12. Raman spectra for **$Bi_{0.90}Eu_{0.10}$-1** collected on a single crystal picked from the bulk material. The peaks between 1750 and 3000 cm-1 show various harmonics of the $^5D_0 \rightarrow {}^7F_1$ transition for Eu^{3+}. The inset highlights the region between 1300 and 2200 cm^{-1} for clarity, as well as an image of the crystal surface prior to data collection.

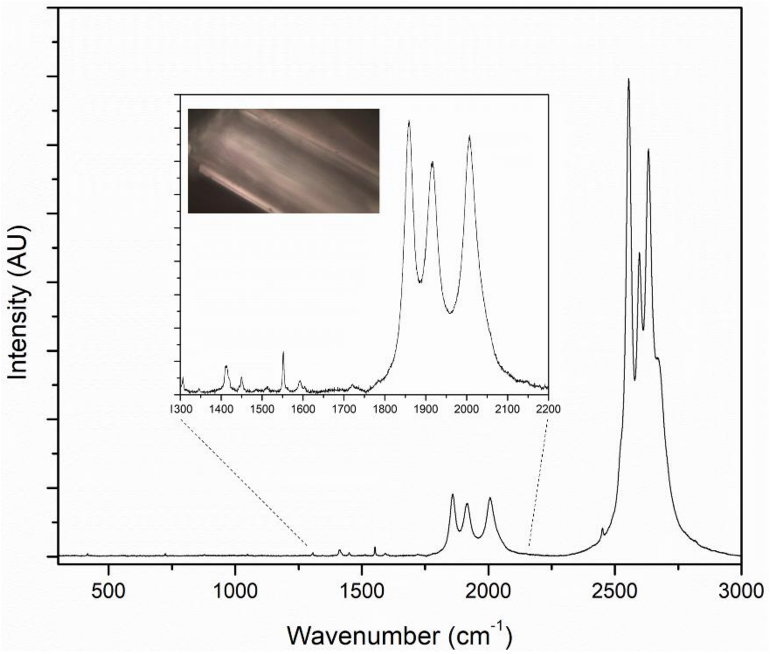

Figure S13. Raman spectra for **$Bi_{0.90}Eu_{0.10}$-1** collected on a single crystal picked from the bulk material, probing focal depth to show Eu^{3+} incorporation inside the crystal, rather than just on the surface. The peaks between 1750 and 3000 cm-1 show various harmonics of the $^5D_0 \rightarrow {}^7F_1$ transition for Eu^{3+}. The inset highlights the region between 1300 and 2200 cm^{-1} for clarity, as well as an image of the crystal surface prior to data collection. The crystal image is blurry as the focal point for data collection has been lowered below the crystal surface.

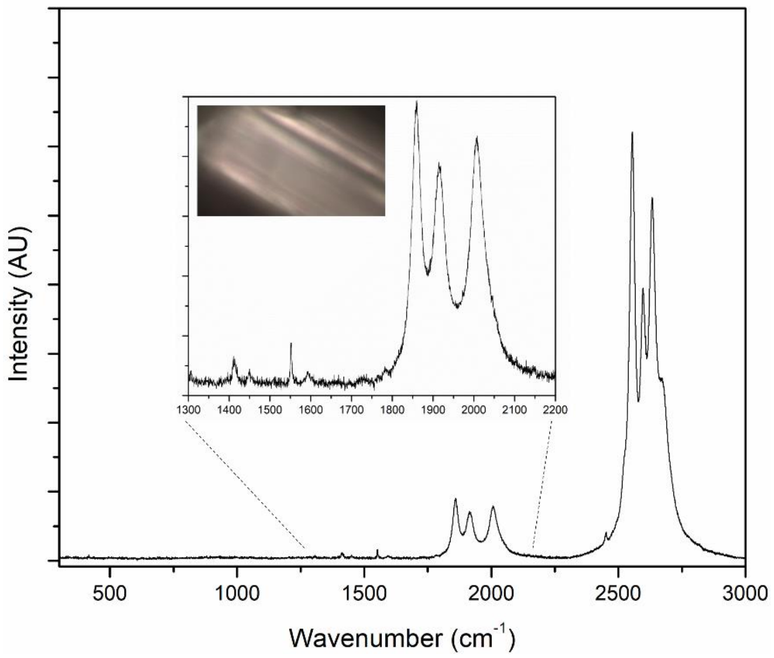

Figure S14. Raman spectra for **$Bi_{0.90}Eu_{0.10}$-1** collected on a single crystal picked from the bulk material, probing focal depth to show Eu^{3+} incorporation inside the crystal, rather than just on the surface. The peaks between 1750 and 3000 cm-1 show various harmonics of the $^5D_0 \rightarrow {}^7F_1$ transition for Eu^{3+}. The inset highlights the region between 1300 and 2200 cm^{-1} for clarity, as well as an image of the crystal surface prior to data collection. The crystal image is blurry as the focal point for data collection has been lowered below the crystal surface, and is lowered further than Figure S13.

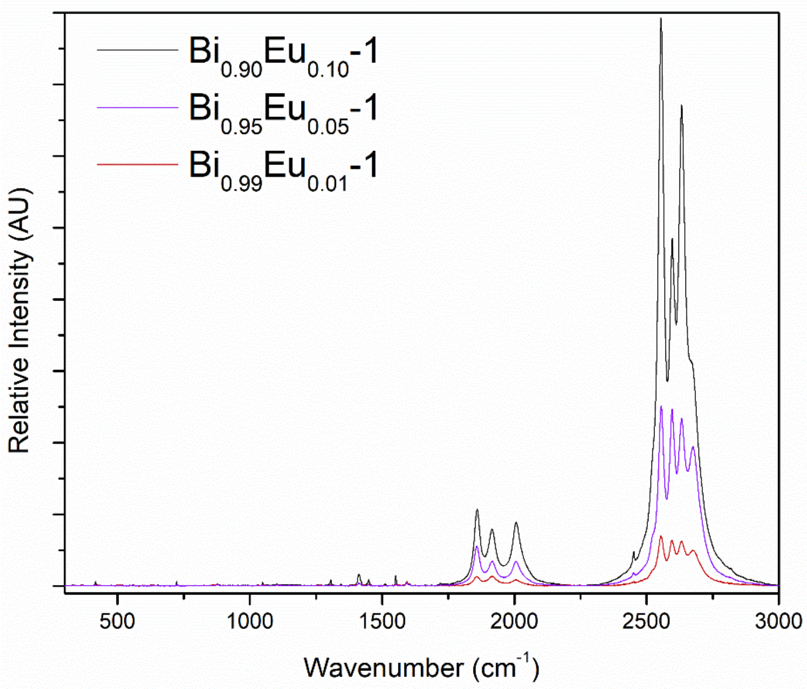

Figure S15. Overlaid Raman spectra for **$Bi_{0.99}Eu_{0.01}$-1** (red), **$Bi_{0.95}Eu_{0.05}$-1** (purple), and **$Bi_{0.90}Eu_{0.10}$-1** (black) collected on single crystals picked from the bulk materials. The peaks between 1750 and 3000 cm-1 show various harmonics of the $^5D_0 \rightarrow {}^7F_1$ transition for Eu^{3+}. Increasing intensity of the Eu^{3+} harmonic peaks with increasing Eu^{3+} concentration within the material is evident.

Table S1. Intensities of peaks at 1593 cm^{-1} (vibrational peak present in **Bi-1**) and 1860 cm^{-1} (Eu^{3+} harmonic peak), as well as the ratio between the two peaks for all Eu-doped Raman spectra. A higher number is consistent with the presence of more Eu^{3+} in the crystal. The consistent ratio between the Bi-organic vibrational peak and the Eu^{3+} harmonic peak in the focal depth experiment for **$Bi_{0.90}Eu_{0.10}$-1** suggests homogeneous incorporation of Eu^{3+} inside the crystal and not just at the surface.

Sample	Intensity @ 1593 cm^{-1} (AU)	Intensity @ 1860 cm^{-1} (AU)	Intensity Ratio ($I_{1860cm-1}$ / $I_{1593cm-1}$)
$Bi_{0.90}Eu_{0.10}$-1 Surface	92.5	2127	23.0
$Bi_{0.90}Eu_{0.10}$-1 Interior #1	37.9	820	21.6
$Bi_{0.90}Eu_{0.10}$-1 Interior #2	12.0	269	22.4
$Bi_{0.95}Eu_{0.05}$-1	78.3	1115	14.2
$Bi_{0.99}Eu_{0.01}$-1	120.8	267	2.21

VI. Photoluminescence Sample Preparation and Data Collection

Spectra were collected on ground, solid samples at room temperature using a slit width of 3 nm for **Bi-1**, and 1 nm for the Eu-doped samples. Excitation wavelengths of 374 nm for **Bi-1** and 350 nm for **$Bi_{0.99}Eu_{0.01}$-1**, **$Bi_{0.95}Eu_{0.05}$-1**, and **$Bi_{0.90}Eu_{0.10}$-1** were utilized. Lifetime measurements were collected with a lamp frequency of 100 Hz, and exponential decay curves were fit with OriginPro 8.5. For the Eu-doped samples, (**$Bi_{0.99}Eu_{0.01}$-1**, **$Bi_{0.95}Eu_{0.05}$-1**, and **$Bi_{0.90}Eu_{0.10}$-1**), a 400 nm long-pass filter was used to avoid harmonic peaks from the excitation source. CIE chromaticity coordinates were calculated in MATLAB from the emission spectra. QY measurements were collected in triplicate using a Horiba PTI QM-400 fluorometer with 1 nm slit widths using a Teflon powder holder under ambient conditions and an 8.9 cm integrating sphere with a Spectralon fluoropolymer coating. The bulk sample of each compound was ground in a mortar and pestle with dry KBr as a matrix prior to QY measurements. Blank absorption and emission spectra were collected using a sample holder filled with dry KBr.

VII. Excitation Spectra for Eu Doped Samples

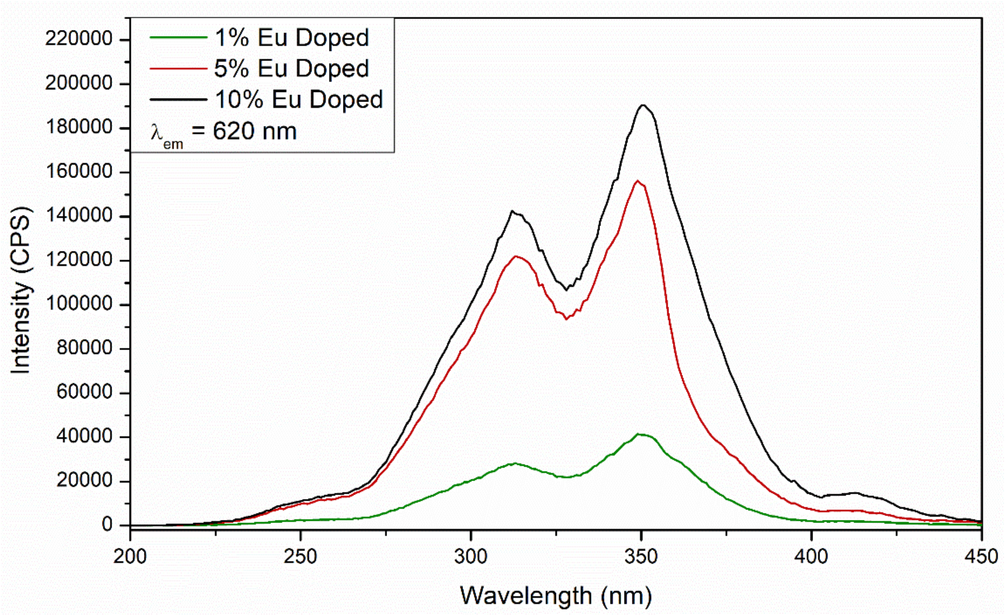

Figure S16. Excitation spectra for **$Bi_{0.99}Eu_{0.01}$-1** (green line), **$Bi_{0.95}Eu_{0.05}$-1** (red line), and **$Bi_{0.90}Eu_{0.10}$-1** (black line). Intensity increases as concentration of Eu^{3+} increases, consistent with emission spectra.

VIII. Lifetime Measurements

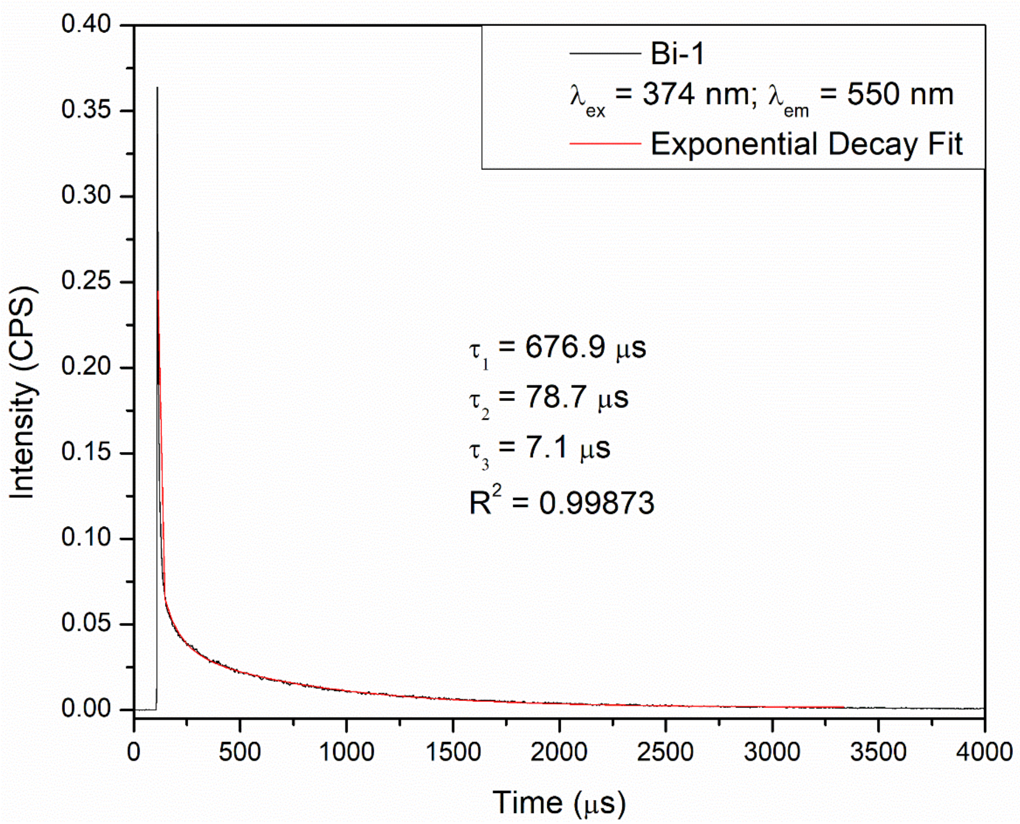

Figure S17. Luminescence decay plot for compound **Bi-1** (black line) fitted with a triple-exponential decay function (red line). Emission recorded at 550 nm upon excitation at 374 nm.

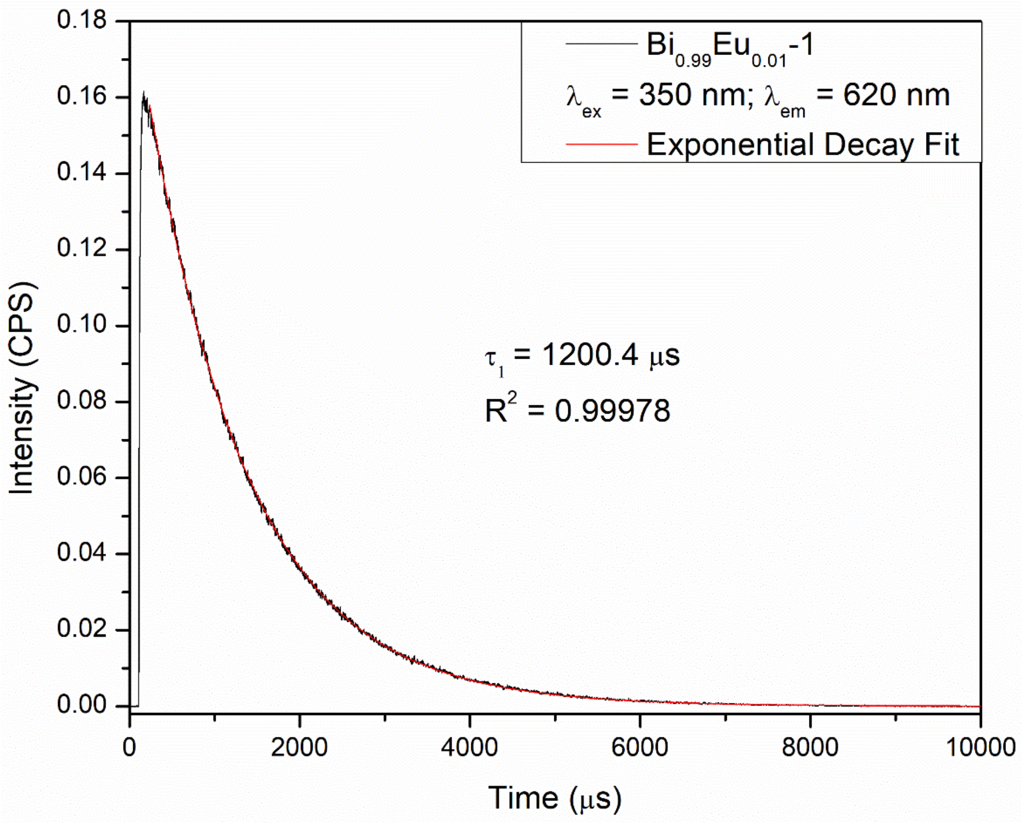

Figure S18. Luminescence decay plot for compound **$Bi_{0.99}Eu_{0.01}$-1** (black line) fitted with a single exponential decay function (red line). Emission recorded at 620 nm upon excitation at 350 nm.

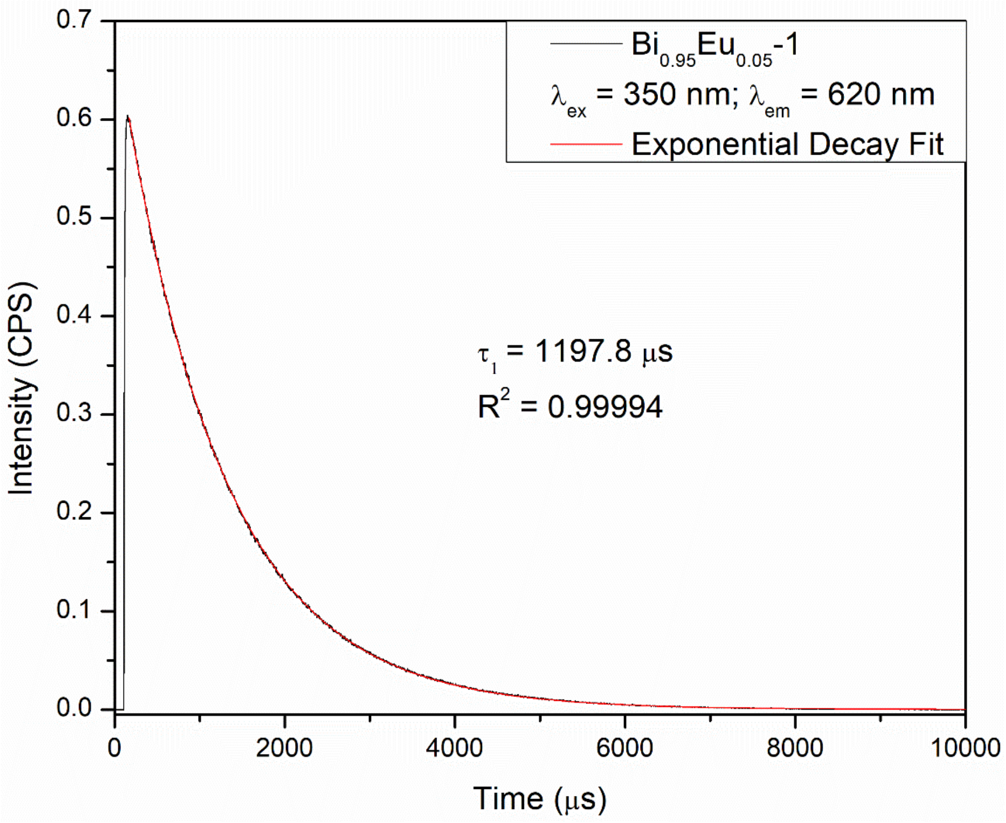

Figure S19. Luminescence decay plot for compound **$Bi_{0.95}Eu_{0.05}$-1** (black line) fitted with a single exponential decay function (red line). Emission recorded at 620 nm upon excitation at 350 nm.

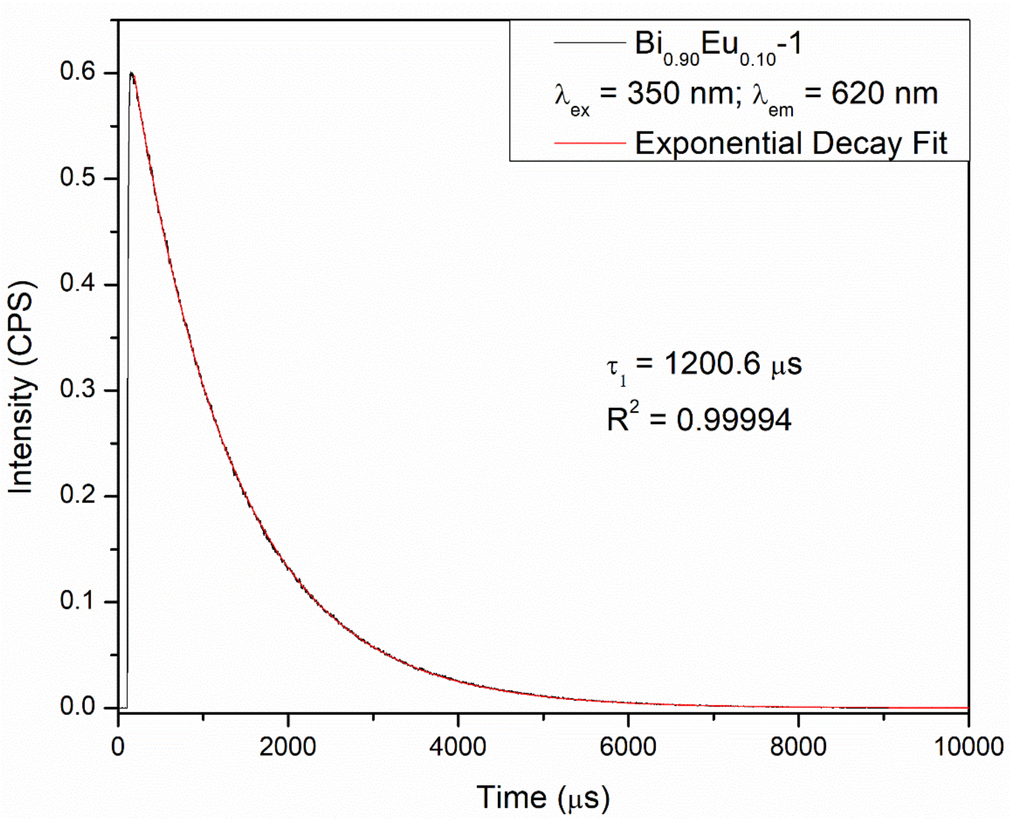

Figure S20. Luminescence decay plot for compound **$Bi_{0.90}Eu_{0.10}$-1** (black line) fitted with a single exponential decay function (red line). Emission recorded at 620 nm upon excitation at 350 nm.

IX. Supramolecular Interactions

Non-covalent interactions including π-π stacking and hydrogen bonding were calculated using the PLATON software suite *via* the "Calc All" feature. These supramolecular interactions are shown in the table below, with appropriate distances/angles as determined by literature precedence.[6-8]

Table S2. Supramolecular interactions for **Bi-1**. C_{gPhen} is one of the centroids formed by the phenanthroline.

Interaction	Distance (Å), C_g---C_g or M---C_g	β (°)	Distance (Å), D-H---A	Angle (°), D-H---A
C_{gPhen}---C_{gPhen}	3.643(1)	22.9	-	-
O(24)-H---O(23)	-	-	2.650(2)	176(10)

X. References

1. *SAINT Bruker AXS Inc.: Madison, WI, USA, 2007*.
2. *APEX3 Bruker AXS Inc.: Madison, WI, USA, 2016.*
3. SADABS. *Bruker AXS Inc.: Madison, WI, USA, 2016.*
4. Hubschle, C. B. S., G. M.; Dittrich, B. ShelXle: a Qt graphical user interface for SHELXL. *J. Appl. Cryst.* **2011,** *44* (6), 1281-1284.
5. Sheldrick, G. A short history of SHELX. *Acta Crystallogr., Sect. A: Found. Adv.* **2008,** *64* (1), 112-122.
6. Janiak, C. A critical account on π–π stacking in metal complexes with aromatic nitrogen-containing ligands. *J. Chem. Soc., Dalton Trans.* **2000**, 3885-3896.
7. Steiner, T. C-H-O Hydrogen Bonding in Crystals. *Crystallogr. Rev.* **1996,** *6*, 1-51.
8. Steiner, T. The Hydrogen Bond in the Solid State. *Angew. Chem. Int. Ed.* **2002,** *41*, 48-76.
